# Exploring Accuracy Limits of Predictions of the ^1^H NMR Chemical Shielding Anisotropy in the Solid State

**DOI:** 10.3390/molecules24091731

**Published:** 2019-05-03

**Authors:** Jiří Czernek, Jiří Brus

**Affiliations:** Institute of Macromolecular Chemistry, Czech Academy of Sciences, Heyrovsky Square 2, 16206 Prague, Czech Republic; brus@imc.cas.cz

**Keywords:** chemical shielding anisotropy, MAS NMR, plane-waves DFT, GIPAW

## Abstract

The ^1^H chemical shielding anisotropy (CSA) is an NMR parameter that is exquisitely sensitive to the local environment of protons in crystalline systems, but it is difficult to obtain it experimentally due to the need to concomitantly suppress other anisotropic interactions in the solid-state NMR (SSNMR) pulse sequences. The SSNMR measurements of the ^1^H CSA are particularly challenging if the fast magic-angle-spinning (MAS) is applied. It is thus important to confront the results of both the single-crystal (SC) and fast-MAS experiments with their theoretical counterparts. Here the plane-waves (PW) DFT calculations have been carried out using two functionals in order to precisely characterize the structures and the ^1^H NMR chemical shielding tensors (CSTs) of the solid forms of maleic, malonic, and citric acids, and of L-histidine hydrochloride monohydrate. The level of agreement between the PW DFT and either SC or fast-MAS SSNMR ^1^H CSA data has been critically compared. It has been found that for the eigenvalues of the ^1^H CSTs provided by the fast-MAS measurements, an accuracy limit of current PW DFT predictions is about two ppm in terms of the standard deviation of the linear regression model, and sources of this error have been thoroughly discussed.

## 1. Introduction

The ^1^H chemical shielding anisotropy (CSA) is expected to be one of the SSNMR parameters that should be particularly useful in describing local structural and dynamical effects experienced by the investigated protons [1]. However, accurate experimental determination of the CSA is difficult (in a typical system, the dipolar ^1^H–^1^H interactions are present which are much stronger than the CSA). Most of the ^1^H CSA data was obtained by analyzing the rotation patterns from the single-crystal (SC) measurements performed with the decoupling of ^1^H–^1^H dipolar interactions [2]. Only relatively recently an important alternative to tedious and obviously limited SC experiments appeared, when it became possible to apply the magic-angle spinning (MAS) SSNMR technique with fast rotation rates to powder samples and reliably assess the ^1^H CSA parameters (several other options are surveyed in Reference [3], which also mentions the solution NMR studies of the ^1^H CSA). The ^1^H CSA data gleaned from this type of the MAS SSNMR measurements during the 2007–ca. mid-2017 period were summarized in Reference [4]. Later, the ultrafast MAS experiments on pharmaceutically active hydrates [5] and small peptides [6] also led to the ^1^H CSA values. It should be noted that above-mentioned SC measurements directly characterize the full ^1^H-NMR chemical shielding tensor, $\sigma^{⃡}$, in terms of its three eigenvalues, $\sigma_{11}$, $\sigma_{22}$, $\sigma_{33}$ ($\sigma_{11}\leq$ $\sigma_{22}\leq \sigma_{33})$, and the eigenvectors, $\vec{\xi_{1}}$, $\vec{\xi_{2}}$, $\vec{\xi_{3}}$, associated with them (the eigenvectors are then used to describe an orientation of $\sigma^{⃡}$ in the reference frame of an investigated crystal). However, the tensorial information from the MAS experiments performed with high spinning rates is available only through fitting of the CSA recoupled line shapes, with the implementation of the underlying theory currently developed in terms of the anisotropy parameter, ζ, $\zeta =\;\sigma_{33}-\sigma^{\mathrm{iso}}$, where $\sigma^{\mathrm{iso}}$ is the isotropic chemical shielding, $\sigma^{\mathrm{iso}}=\frac{1}{3\left(\sigma_{11}+\sigma_{22}+\sigma_{33}\right)}$, and of the asymmetry factor, η, $\eta =\frac{\left(\sigma_{22}-\sigma_{11}\right)}{\zeta }$. An evaluation of the simulated spectra yields only an absolute value, $\left|\zeta \right|$, of the anisotropy ζ (due to symmetry properties of the involved Hamiltonian [7]), while uncertainty in the η data can sometimes be quite large (several tenths of a value which is between 0 and 1, see Table 2 in Reference [3]). It is thus of keen interest to employ the results of the MAS and of generally more reliable SC SSNMR measurements and to establish accuracy limits of state-of-the-art quantum chemical methods when applied to the prediction of the ^1^H CSA in molecular crystals. This confrontation of theory with two types of experiments not only provides an assessment of the computational methods, but it also enables to address possible uncertainties in the MAS SSNMR results. In this investigation, the plane-wave (PW) density functional theory (DFT) is combined with the gauge-including projector augmented-wave (GIPAW) [8,9] method in order to reproduce two types of experimental data. The first type concerns the CSA of protons in maleic and malonic acids accurately characterized by the SC SSNMR experiments long ago by Haeberlen et al. [10,11]. The second type pertains to the frequently studied L-histidine hydrochloride monohydrate, of which precisely measured isotropic {^1^H, ^13^C, ^15^N} [12] and ^15^N anisotropic [13] data are considered together with the ^1^H CSA from the MAS experiments [3,14]. In addition, the ^1^H CSA information obtained for citric acid from the ultrafast MAS three-dimensional (3D) correlations is addressed [15].

This analysis of accuracy limits of the PW DFT technique is the initial step towards incorporation of the ^1^H CSA data into the NMR crystallography approaches for structural elucidation/refinement of compounds in the condensed phase [16]. It is easy to envision investigations similar to those which very recently adopted the ^13^C [17,18,19,20], ^15^N [21] or ^31^P [22] CSA in NMR crystallography studies. Moreover, since the eigenvalues of the ^1^H chemical shielding tensors can be particularly sensitive to structure, they could potentially be employed in methods for selecting the suitable candidate(s) from among the generated crystal structure predictions [23,24,25,26].

## 2. Results

### 2.1. Comparison of the DFT and SC SSNMR Data

**Figure 1:**
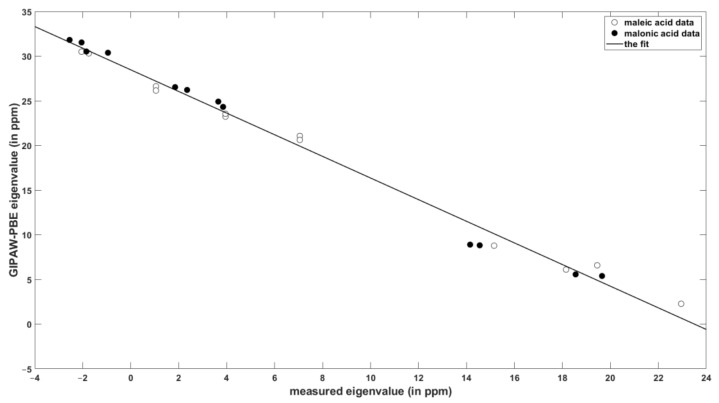
The correlation of the calculated and experimental values of the principal elements of the ^1^H chemical shielding/shift tensors in maleic and malonic acids.

The results of painstaking SC measurements of the eigenvalues of the ^1^H chemical shielding tensors and their orientations in the crystal frame of maleic and malonic acids [10,11] served as the reference data against which the performance of the PW DFT calculations was checked. Table 1 summarizes the key statistical parameters describing the level of agreement between theory and experiment (the raw values are gathered in the Supplementary Information, Tables SI1 and SI5). The values of the slope and intercept of the linear relationship between the chemical shielding and chemical shift data are similar for the set of isotropic values and for the principal components of the ^1^H tensors. The errors are small for the isotropic chemical shielding/shift, as expected [27,28], and they increase to only about one ppm of the standard deviation for the eigenvalues (see Table 1), while it should be noted that the corresponding measurement uncertainties were estimated to be ± 0.5 ppm. The linear regression model of the principal elements is graphically presented in Figure 1.

The spatial orientation of the ^1^H chemical shielding tensors of all four protons of malonic acid [11], and of the two protons involved in hydrogen bonding in maleic acid [10], was established experimentally. In each case an orientation of these tensors in the molecular frame follows the pattern of (1) the most shielded component almost collinear with the H–X bond vector (X is either O or C); (2) the mid-shielded component approximately perpendicular to the H–X bond while lying close to a plane formed by the H, X, and the nearest heteroatom; and (3) the least shielded component in the direction approximately perpendicular to that plane. This pattern was confirmed by the PW DFT calculations and is analyzed in detail in the Supplementary Information in terms of the reference vectors associated with the local geometry of the hydrogens of malonic acid. Regarding numerical values, Haeberlen et al. reported for all the carboxylic protons of malonic and maleic acids an angle of 8° ± 2° between the most shielded direction of the ^1^H chemical shielding tensor and the line between the oxygens of the corresponding hydrogen bond [10]. This result is in good agreement with the theoretical predictions of 7°, 9°, 5°, and 12° obtained using the PBE functional respectively for protons denoted as H1 and H2 in both structures [29,30]. There is only a mild dependence of these values on the choice of the DFT functional: They accordingly amount to 8°, 11°, 4°, and 13° when computed with the revPBE. In addition, the parameters of the theory-to-experiments fits are fairly similar for the two functionals (see Table 1). Thus, the periodic DFT computations can be expected to quantitatively reproduce the results of the SC ^1^H SSNMR measurements of the chemical shielding tensors’ orientations, and to provide the eigenvalues with the root-mean-squared error (RMSE) of about 1.0 ppm in the linear regression model.

### 2.2. The {^13^C, ^15^N, ^1^H} Results for Biprotonated l-Histidine

The statistical data collected in Table 2 and Table 3 are discussed below.

**Figure 2:**
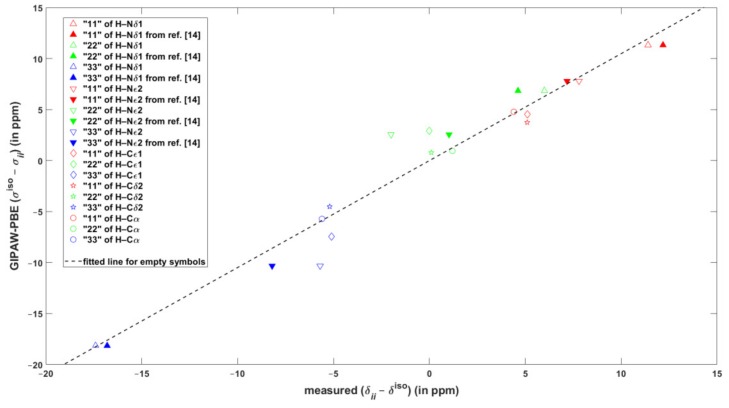
The correlation of the calculated and experimental differences between the values of the isotropic chemical shift/shielding and of the respective principal element of the ^1^H chemical shift/shielding tensors in L-histidine hydrochloride monohydrate.

The performance of the GIPAW calculations of the chemical shielding was carefully evaluated also for the major tautomer of L-histidine, which was frequently studied in the SSNMR experiments [12], and of which neutron diffraction structure is available [31]. In this system, the ^1^H and ^13^C isotropic chemical shift values span large intervals of more than 13 and almost 150 ppm, respectively (see SI Tables SI2 and SI3). As follows from an inspection of Table 2, the agreement of the DFT predictions with these isotropic data can be considered to be very good. For example, the maximum regression errors are just about 0.3 and 2 ppm accordingly for ^1^H and ^13^C results. The principal elements of the ^15^N chemical shift tensors and their orientation in the crystal frame as obtained from the SC measurements [13] are also reliably reproduced by the calculations (see Table 2 and Table SI7, respectively). However, as for the ^1^H chemical shielding anisotropies inferred from the fast MAS measurements of powdered samples [3,14], the agreement between theory and experiment is less satisfactory. It is emphasized that in Reference [3] the traceless chemical shift tensors were reported, that is, the results were presented in terms of differences $\left(\delta_{ii}-\delta^{\mathrm{iso}}\right)$ between a principal component of the chemical shift tensor, $\delta_{ii}$, and the corresponding isotropic chemical shift, $\delta^{\mathrm{iso}}$. For the sake of comparison with the experiments, $\left(\sigma^{\mathrm{iso}}-\sigma_{ii}\right)$ data are considered here. Once the $\left(\sigma^{\mathrm{iso}}-\sigma_{ii}\right)$ differences are fitted to their $\left(\delta_{ii}-\delta^{\mathrm{iso}}\right)$ counterparts, the values of the slope and intercept are very close to unity and to zero ppm, respectively (such a fit is graphically presented in Figure 2, while the statistical parameters are gathered in Table 3 for both GIPAW-PBE and GIPAW-revPBE results). It is immediately seen from Figure 2 that the calculations reliably reproduced the large span of the $\left(\delta_{ii}-\delta^{\mathrm{iso}}\right)$ differences, which amounts to about 29 ppm. Figure 2 also illustrates rather high (approaching 5 ppm) regression errors for data of the protons bound to imidazole nitrogens of l-histidine hydrochloride monohydrate. Importantly, for those two sites, the ^1^H CSA data were extracted from the two-dimensional (2D) ^1^H CSA/^1^H CS (the isotropic chemical shift) correlations recorded under ultrafast MAS [14] and can be used to illustrate a pronounced sensitivity of the ^1^H CSA parameters to various experimental factors. Thus, the CS, ζ, and η values reported in Reference [14] were converted to the $\left(\delta_{ii}-\delta^{\mathrm{iso}}\right)$ differences and are depicted as filled symbols in Figure 2. The apparent scatter in experimental values should be compared with much smaller uncertainties in the results of the SC measurements. While it is noted that the GIPAW predictions agree much better with the SC (see Section 2.1) than with the fast MAS results, it appears that the errors are significantly affected by inaccuracies of the MAS measurements caused by distortions of CSA line shapes. It should also be noted that even the CS value of the Hδ1 proton differs between measurements in References [12] and [14] (respectively amounting to 17.1 and 16.8 ppm), while it is the same in case of Hε2 (12.6 ppm). This further illustrates inherent difficulties in properly establishing the level of agreement between theory and experiment in ^1^H SSNMR (see Reference [27] for discussion).

### 2.3. The ^1^H CSA in Citric Acid

^1^H chemical shielding tensors of the four protons involved in hydrogen bonding in crystalline citric acid [32]. These tensors were studied by the 3D correlation experiments performed under ultrafast MAS [15], and it should be mentioned that their ^1^H CSA parameters were previously obtained from the 2D experiments [7,33], and also that they exhibited significant uncertainties in the η values. The CS, ζ, and η data were taken from Table 1 of Reference [15] and converted to the values of the principal elements, which were then compared to their theoretical counterparts. Table 4 presents an evaluation of the linear regressions (raw data are collected in Table SI6). For the periodic DFT calculations, the RMSE is about 1.7 ppm, but this value might be strongly affected by experimental uncertainties, as in the case of correlations for l-histidine hydrochloride monohydrate discussed above. 

**Figure 3:**
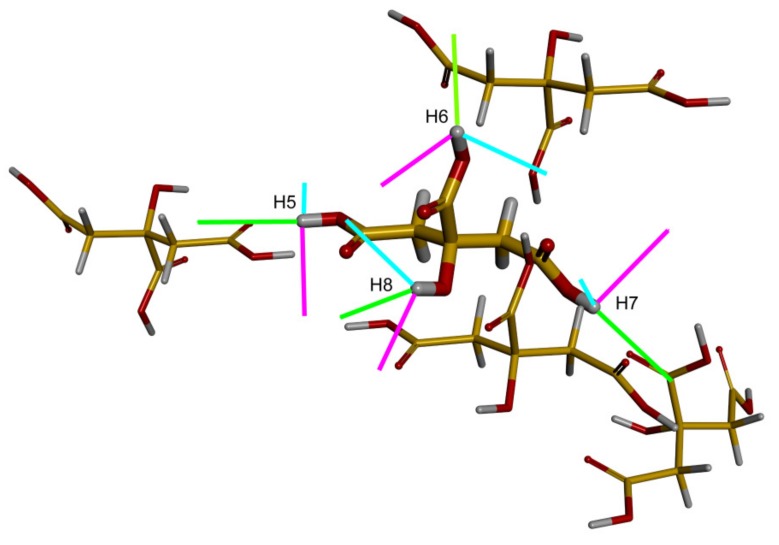
The molecular cluster of citric acid. In the central molecule, an orientation of the eigenvectors of the ^1^H chemical shielding tensors is shown (the eigenvectors associated with the smallest, mid, and highest eigenvalues are colored in magenta, cyan, and green, respectively).

The most challenging case for computational methods considered here is that of the The results are less accurate for the cluster model, depicted in Figure 3, with the RMSE of about 2.5 ppm and the maximal error exceeding 5 ppm (see Table 4). However, this cluster was created mainly in order to check the orientation of the respective ^1^H chemical shielding tensors in the molecular frame (to avoid any possible confusion related to an orientation in the *P*2_1_/*n* crystal frame of citric acid in CASTEP calculations), as this spatial information should be useful in simulations of the 2D ^1^H CSA/^1^H CSA correlations [15]. It was, nevertheless, established that the results of the approach used in the cluster calculations, GIAO-B3LYP/6-311++G(2d, 2p), should be of a quality similar to the GIAO-MP2/6-311++G(2d, 2p), at least for hydrogen-bonded models (see Table SI10 showing the variation of the ^1^H-NMR parameters in the phenol–water dimer). It was also verified that essentially the same orientations in the crystal/molecular frame were provided by the GIPAW-PBE and the cluster calculations for the investigated ^1^H chemical shielding tensors in citric acid (Tables SI8 and SI9). These orientations follow the pattern described for malonic acid in Part 2.1 and are shown in Figure 3. In particular, it should be realized that the angle is small between the O–H bond vector and the direction of $\vec{\xi_{3}}$, the eigenvector associated with the most shielded component, $\;\sigma_{33}$. It should also be realized that the hydrogen bond direction can be roughly approximated by a vector between the acceptor and donor oxygens. This vector is denoted here as $\vec{h}\left(k\right)$, $\vec{h}\left(k\right)=\vec{\left|\mathrm{Oa}\left(Hk\right)-\mathrm{Od}\left(Hk\right)\right|}$, where $\left(Hk\right)$ relates to one of the {H5, H6, H7, H8} protons of citric acid, and an angle $\varphi \left(i,\;j\right)$ between the two vectors $\vec{h}\left(i\right),\;\vec{h}\left(j\right)$ describes a mutual orientation of the hydrogen bonds involving Hi and Hj. Let us further define $\psi \left(Hi,\;Hj\right)$ as an angle between the $\vec{\xi_{3}}$ of one of the {H5, H6, H7, H8} protons and the hydrogen bond involving any proton from this set. For example, $\psi \left(H5,\;H6\right)$ is an angle between $\vec{\xi_{3}}$ of H5 and $\vec{h}\left(6\right)$, which is the vector of the hydrogen bond associated with H6. Then for each Hi, values of $\psi \left(Hi,\;Hj\right)$ angles are expected to be close to corresponding $\varphi \left(i,\;j\right)$ values. This indeed holds for H5, as exemplified in Table 5 (the GIAO-B3LYP/6-311++G(2d, 2p) chemical shielding and the PW PBE coordinates were used to obtain the $\varphi \left(5,\;j\right)$ and $\psi \left(H5,\;Hj\right)$ data). Hence, angles between hydrogen bonds would be quite accurately described by relative orientations of the ^1^H chemical shielding tensors of protons involved in the hydrogen bonding. These relative orientations are available from cross-correlations in the 2D ^1^H CSA/^1^H CSA spectra [15].

## 3. Discussion

As noted in the introduction, there is an increasing interest in measurements of the ^1^H CSA by recoupling the anisotropic interactions under fast MAS frequencies. The respective experimental approaches (namely, the heteronuclear-detected [34] or proton-detected [7,35] techniques) have inherent limitations in the reliability of the extracted parameters of the ^1^H chemical shielding tensors. Some of the issues (a correct sign of the anisotropy parameter ζ, an orientation in the crystal frame of the principal axis systems of investigated tensors) can be directly addressed by the PW DFT calculations. Moreover, those calculations are expected to play a crucial role in a structural interpretation of the results of the above-mentioned experiments. Therefore, it is important to find accuracy limits of computational protocols, which are already in use for other parameters in the NMR crystallography framework, when applied to the predictions of the ^1^H CSA data for static structures. When the SC values of the principal components of the chemical shielding tensors of protons in maleic and malonic acids are taken as the reference, there is about 1.0 ppm RMSE in their theoretical (GIPAW-PBE and GIPAW-revPBE) counterparts. This benchmark value reflects not only limitations of the computations (mainly due to deficiencies in the DFT functionals and neglect of external effects, the temperature in particular [36,37,38]), but also ±0.5 ppm uncertainty in the measured data [10,11]. Nonetheless, using the CSA data from fast MAS studies of L-histidine hydrochloride monohydrate [3,14] and citric acid [15], the RMSE becomes about two times higher. This increase reflects inaccuracies in value of the asymmetry parameter η and dependence of the experimental results on the actual choice of the CSA recoupling sequence. As for the spatial orientation of the ^1^H chemical shielding tensors, the PW DFT calculations reproduce the data from SC measurements reliably, with only several-degrees differences. It is shown that it is possible to describe relative arrangements of hydrogen bonds simply by following an angle between the direction of the most shielded component of the ^1^H chemical shielding tensor of two pertinent protons (see Section 2.3 for details), and that this information can also be reliably obtained from the GIAO-B3LYP calculation performed with a saturated basis for a cluster model (of course assuming the overall structure is described correctly).

## 4. Materials and Methods

The PW DFT computational approach, which directly includes crystal-lattice effects upon the investigated parameters of solids (see References [39,40,41] for details), was used as implemented in the CASTEP version 16.1 program package [41]. The coordinates of the *P2*_1_/*c* polymorph of maleic acid [29], the *β* polymorph of malonic acid [30], and the *P2*_1_/*n* structure of citric acid [32] were taken from the XRD studies, and the coordinates of the neutron diffraction structure were considered in case of l-histidine hydrochloride monohydrate [31]. They served as input for the minimizations of the lattice energy with respect to all internal coordinates, with unit cell parameters fixed at experimental values. Both the PBE [42] and the revPBE (‘revised PBE’) [43] DFT exchange-correlation functionals were employed together with the CASTEP settings consistent with the ‘Fine’ level of accuracy of the Materials Studio 5.0 software (the technical assistance was provided by Dr. M. Hušák, University of Chemistry and Technology, Prague). In particular, the cut-off value of 550 eV for the plane-waves energy was applied, and the Monhorst–Pack grids [44] for maleic acid, malonic acid, citric acid, and L-histidine hydrochloride monohydrate calculations were respectively 4 × 2 × 4, 8 k-points; 7 × 5 × 3, 53; 2 × 4 × 2, 4; and 2 × 3 × 4, 4. The default on-the-fly generation of ultrasoft pseudopotentials was adopted. For the structures thus obtained, the corresponding GIPAW-PBE and GIPAW-revPBE chemical shielding tensors were predicted in the CASTEP NMR module while also using the ‘Fine’ settings. Molecular complexes were studied using the Gaussian 09 suite of quantum chemical programs [45]. The citric acid cluster was investigated at the standard B3LYP/6-311++G(2d, 2p) level with the GIAO (gauge-independent atomic orbitals) [46,47] technique applied to overcome the gauge problem of chemical shielding calculations. The atomic coordinates of this cluster were generated from the periodic structure optimized using the PBE functional. The model of phenol–water dimer was prepared using interactive computer graphics (Insight II (2000), Accelrys Inc., San Diego, CA, USA) approximately in an arrangement of ‘Structure 1′ from Reference [48], and its potential energy minimum was located at the standard MP2/aug-cc-pVTZ level. This structure was then used for an unrelaxed scan of the ^1^H chemical shielding tensor of phenolic hydrogen. Namely, the distance between the two oxygens was varied in the interval from 261 to 351 pm, and at each point, both the GIAO-MP2 and GIAO-B3LYP chemical shielding was predicted with the 6-311++G(2d, 2p) basis set. The eigenvectors of the ^1^H chemical shielding tensors were processed by the INFOR software [49] for visualization.

## Figures and Tables

**Table 1:** Statistical evaluation of the agreement between the GIPAW-PBE (in parentheses, the GIPAW-revPBE) chemical shieldings and experimental chemical shifts for protons of maleic and malonic acids.

Parameter	Isotropic	Eigenvalues
slope	–1.255	–1.211
	(–1.194)	(–1.159)
standard error of slope	0.034	0.026
	(0.030)	(0.024)
intercept/(ppm)	28.78	28.47
	(28.88)	(28.64)
standard error of intercept/(ppm)	0.28	0.28
	(0.25)	(0.26)
standard deviation/(ppm)	0.39	1.03
	(0.34)	(0.96)
average absolute deviation/(ppm)	0.30	0.81
	(0.30)	(0.77)
maximum absolute deviation/(ppm)	0.73	2.43
	(0.55)	(2.26)
adjusted *R^2^*	0.9949	0.9895
	(0.9956)	(0.9899)
number of data points	8	24
	(8)	(24)

**Table 2:** Statistical evaluation of the agreement between the GIPAW-PBE (in parentheses, the GIPAW-revPBE) {^1^H, ^13^C, ^1^N} chemical shielding data and their experimental counterparts for l-histidine hydrochloride monohydrate.

Parameter	^1^H isotropic	^13^C isotropic	^15^N eigenvalues
slope	–1.108	–1.019	–1.047
	(–1.121)	(–1.016)	(–1.057)
standard error of slope	0.019	0.010	0.028
	(0.020)	(0.010)	(0.032)
intercept/ (ppm)	31.43	171.72	206.5
	(31.80)	(171.71)	(204.8)
standard error of intercept/(ppm)	0.19	1.23	5.2
	(0.19)	(1.22)	(5.9)
standard deviation/(ppm)	0.21	1.15	5.6
	(0.21)	(1.15)	(6.4)
average absolute deviation/(ppm)	0.16	0.76	4.6
	(0.19)	(0.73)	(5.3)
maximum absolute deviation/(ppm)	0.30	2.15	7.8
	(0.26)	(2.16)	(9.6)
adjusted *R^2^*	0.9982	0.9995	0.9964
	(0.9982)	(0.9995)	(0.9954)
number of data points	7	6	6
	(7)	(6)	(6)

**Table 3:** Statistical evaluation of the agreement between the theoretical $\left(\sigma^{\mathrm{iso}}-\sigma_{ii}\right)$ differences and their experimental $\left(\delta_{ii}-\delta^{\mathrm{iso}}\right)$ counterparts for the ^1^H sites in cationic l-histidine (15 data points).

Parameter	GIPAW-PBE	GIPAW-revPBE
slope	1.049	1.053
standard error of slope	0.079	0.080
intercept/(ppm)	–0.007	–0.007
standard error of intercept/(ppm)	0.550	0.554
standard deviation/(ppm)	2.05	2.07
average absolute deviation/(ppm)	4.65	4.63
maximum absolute deviation/(ppm)	1.36	1.38
adjusted *R^2^*	0.9258	0.9249

**Table 4:** Statistical evaluation of the agreement between predicted eigenvalues of the ^1^H chemical shielding tensors and experimentally established eigenvalues of the ^1^H chemical shift tensors of the four proton sites in citric acid (12 data points).

Parameter	GIPAW-PBE	GIPAW-revPBE	GIAO-B3LYP
slope	–1.151	–1.095	–1.183
standard error of slope	0.052	0.048	0.075
intercept/(ppm)	30.23	30.56	31.31
standard error of intercept/(ppm)	0.75	0.69	1.07
standard deviation/(ppm)	1.77	1.63	2.54
average absolute deviation/(ppm)	1.34	1.22	1.92
maximum absolute deviation/(ppm)	3.15	2.97	5.20
adjusted *R^2^*	0.9779	0.9793	0.9580

**Table 5:** Angles between vectors in citric acid that are discussed in the text.

Direction	$\vec{\left|O6a-O6d\right|}$	$\vec{\left|O7a-O7d\right|}$	$\vec{\left|O8a-O8d\right|}$
$\vec{\left|O5a-O5d\right|}$	47°	118°	74°
$\vec{\xi_{3}}$ of H5	56°	125°	77°

## Supplementary Materials

The following are available online in ‘SI.pdf’ file: raw data for Table 1, Table 2, Table 3 and Table 4 of the main text, details of the orientation of the imidazole ^15^N chemical shielding tensors in biprotonated l-histidine, comparisons of the ^1^H chemical shielding tensor orientations of citric acid in crystal/molecular frames, the distance-dependence of the ^1^H NMR data in the phenol–water dimer, and projections of the ^1^H chemical shielding tensors of malonic acid onto the molecular frame.
