# Supplementary material for: Exploring Accuracy Limits of Predictions of the 1H NMR Chemical Shielding Anisotropy in the Solid State
Source: Molecules. 2019 May 3;24(9):1731. doi: 10.3390/molecules24091731 (PMC6539467; doi:10.3390/molecules24091731)

Supplementary Information for "Exploring Accuracy Limits of Predictions of the ^{1}H NMR Chemical Shielding Anisotropy in the Solid State" by Czernek and Brus (2019)

Content (ten pages in total):

raw data for Table 1 of the main text … pages 2 (isotropic) and 3 (eigenvalues)

raw data for Table 2 of the main text … pages 2 (isotropic) and 3 (eigenvalues)

raw data for Table 3 of the main text … page 4

details of the orientation of the imidazole ^{15}N chemical shielding tensors … page 4

comparisons of the ^{1}H chemical shielding tensor orientations of citric acid in crystal/molecular frames … page 5

distance-dependence of the ^{1}H NMR data the in the phenol–water dimer … page 6

projections of the ^{1}H chemical shielding tensors of malonic acid onto the molecular frame … pages 7 – 10

Table SI1. The ^{1}H isotropic chemical shift/shielding data (in ppm) for maleic and malonic acids.

site	exptl chemical shift as used here**	GIPAW-PBE σ^{iso}	GIPAW-revPBE σ^{iso}
H1 in maleic acid	10.95	15.2260	15.9568
H2 in maleic acid*	4.0167	23.6430	23.8516
H3 in maleic acid*	4.0167	23.4383	23.6941
H4 in maleic acid	13.0167	12.9602	13.6646
H1 in malonic acid	10.7167	15.2486	16.2169
H2 in malonic acid	10.05	15.4284	16.3289
H3 in malonic acid	1.3833	27.2185	27.5132
H4 in malonic acid	1.5833	27.0794	27.2931

* H2, H3 experimentally unresolved

** after conversion from data referenced to adamantane in references **[10]** and **[11]** of the main text

Table SI2. The ^{1}H isotropic chemical shift/shielding data (in ppm) for L-histidine hydrochloride monohydrate.

site	exptl chemical shift	GIPAW-PBE σ^{iso}	GIPAW-revPBE σ^{iso}
H bound to Cα	3.5	27.7943	28.1004
two* H bound to Cβ	3.3	27.5628	27.8429
H bound to Cε	9.3	21.1359	21.2404
H bound to Cδ	8.0	22.8031	23.0594
H(ammonium)	8.6	21.5953	22.0039
H bound to Nδ	16.8	12.8670	12.8656
H bound to Nε	12.6	17.4304	17.8841

* experimentally unresolved

Table SI3. The ^{13}C isotropic chemical shift/shielding data (in ppm) for L-histidine hydrochloride monohydrate.

site	exptl chemical shift	GIPAW-PBE σ^{iso}	GIPAW-revPBE σ^{iso}
C′	173.2	–5.6730	–2.6127
Cα	54.1	116.4630	117.2207
Cβ	26.0	145.0781	145.6808
Cγ	128.7	39.5322	41.7607
Cδ	136.3	34.9954	37.3542
Cε	119.4	50.2050	52.1428

Table SI4. The principal components (in ppm) of ^{15}N chemical shift/shielding tensors in L-histidine hydrochloride monohydrate (xx, yy, zz respectively denote the most shielded, the mid-shielded, and the least shielded eigenvalue).

site	exptl δ_{ii}	GIPAW-PBE σ_{ii}	GIPAW-revPBE σ_{ii}
xx of Nδ	38.8	167.3731	165.0983
yy of Nδ	198.1	–8.5969	–10.7169
zz of Nδ	260.5	–62.8181	–65.8502
xx of Nε	35.1	172.6674	171.852
yy of Nε	170.1	22.3478	15.4684
zz of Nε	251.3	–50.2537	–54.7112

Table SI5. The principal components (in ppm) of ^{1}H chemical shift/shielding tensors in maleic and malonic acids (xx, yy, zz respectively denote the least shielded, the mid-shielded, and the most shielded eigenvalue).

component, site	exptl** δ_{ii}	GIPAW-PBE σ_{ii}	GIPAW-revPBE σ_{ii}
xx of H1 in maleic acid	19.45	6.5824	7.6042
yy of H1 in maleic acid	15.15	8.7812	9.8148
zz of H1 in maleic acid	–1.75	30.3144	30.4515
xx of H2 in maleic acid*	7.05	21.0694	21.332
yy of H2 in maleic acid*	3.95	23.2329	23.4028
zz of H2 in maleic acid*	1.05	26.6266	26.8199
xx of H3 in maleic acid*	7.05	20.6229	20.9715
yy of H3 in maleic acid*	3.95	23.5430	23.675
zz of H3 in maleic acid*	1.05	26.1491	26.4359
xx of H4 in maleic acid	22.95	2.2770	3.3128
yy of H4 in maleic acid	18.15	6.1046	7.1191
zz of H4 in maleic acid	–2.05	30.499	30.562
xx of H1 in malonic acid	19.65	5.3874	7.0026
yy of H1 in malonic acid	14.55	8.8215	9.9064
zz of H1 in malonic acid	–2.05	31.5369	31.7418
xx of H2 in malonic acid	18.55	5.5659	7.0737
yy of H2 in malonic acid	14.15	8.8986	9.9876
zz of H2 in malonic acid	–2.55	31.8206	31.9255
xx of H3 in malonic acid	3.65	24.9135	25.2372
yy of H3 in malonic acid	2.35	26.2209	26.4656
zz of H3 in malonic acid	–1.85	30.5212	30.8368
xx of H4 in malonic acid	3.85	24.3205	24.6039
yy of H4 in malonic acid	1.85	26.5351	26.6388
zz of H4 in malonic acid	–0.95	30.3826	30.6367

* H2, H3 experimentally unresolved

** after conversion from data referenced to adamantane in references **[10]** and **[11]** of the main text

Table SI6. The principal components (in ppm) of ^{1}H chemical shift/shielding tensors in citric acid (*xx*, *yy*, *zz* respectively denote the least shielded, the mid-shielded, and the most shielded eigenvalue).

component, site	exptl δ_{ii}*	GIPAW-PBE σ_{ii}	GIPAW-revPBE σ_{ii}	GIAO-B3LYP σ_{ii}
xx of H5	22.2	2.3769	3.9860	–0.1642
yy of H5	22.2	5.9180	7.0273	9.7398
zz of H5	–2.7	33.2971	33.4162	34.9793
xx of H6	18.825	9.3172	10.4805	10.2952
yy of H6	15.075	11.8739	13.3139	12.5348
zz of H6	–1.8	32.6174	32.8246	35.0316
xx of H7	17.5	7.2983	9.0027	8.0095
yy of H7	15.0	12.7725	14.5510	13.2200
zz of H7	–2.5	31.8768	31.7619	33.4513
xx of H8	13.54	17.2049	18.0584	16.6294
yy of H8	10.86	20.8799	21.6416	20.5776
zz of H8	–7.9	38.8302	38.9283	39.0381

* Haeberlen notation was used in reference **[15]** of the main text

Table SI7. The orientation of the ^{15}N chemical shielding tensors of the Nδ and Nε sites in L-histidine hydrochloride monohydrate (the *xyz* coordinate system is as described in the caption of Figure 3 of reference **[13]** of the main text, and *xx*, *yy*, *zz* respectively denote the least shielded, the mid-shielded, and the most shielded eigenvalue).

angle	exptl	GIPAW-PBE
between *z* and the eigenvector associated with *xx* of Nδ	0.0°	1.3°
between *x* and the eigenvector associated with *zz* of Nδ	14.2°	16.5°
between *y* and the eigenvector associated with *yy* of Nδ	14.2°	16.5°
between *x* and the Nδ–Hδ bond vector	5.0°	1.8°
between *z* and the eigenvector associated with *xx* of Nε	0.7°	1.5°
between *x* and the eigenvector associated with *zz* of Nε	13.6°	13.7°
between *y* and the eigenvector associated with *yy* of Nε	13.7°	13.8°
between *x* and the Nε–Hε bond vector	2.0°	3.8°

Table SI8. Angles (in degrees) between the eigenvectors associated with the most shielded eigenvalue of {H5, H6, H7, H8} protons in citric acid discussed in the main text (values in upper triangle are from the GIAO-B3LYP/6-311++G(2d,2p) calculation on the cluster model, values in lower triangle: GIPAW-PBE for the periodic structure).

cluster / periodic	$\overrightarrow{\xi_3}$ of H5	$\overrightarrow{\xi_3}$ of H6	$\overrightarrow{\xi_3}$ of H7	$\overrightarrow{\xi_3}$ of H8
$\overrightarrow{\xi_3}$ of H5	0/0	45.6	52.7	84.3
$\overrightarrow{\xi_3}$ of H6	46.2	0/0	88.2	71.0
$\overrightarrow{\xi_3}$ of H7	48.6	86.3	0/0	73.9
$\overrightarrow{\xi_3}$ of H8	87.0	73.0	71.1	0/0

Table SI9. Angles (in degrees) between the eigenvectors associated with the most shielded eigenvalue of {H5, H6, H7, H8} protons and the corresponding O–H bond vector in citric acid discussed in the main text (the GIAO-B3LYP/6-311++G(2d,2p) results were obtained for the cluster model, the GIPAW-PBE for the periodic structure).

site, k	angle between $\overrightarrow{|\mathrm{O}k - \mathrm{H}k|}$ and $\overrightarrow{\xi_3}$ of Hk	
	GIPAW-PBE	B3LYP-GIAO
5	12.4	10.8
6	19.8	15.4
7	19.9	23.7
8	16.6	13.2

Table SI10. The scan of the ^1H NMR parameters of the phenolic proton in the phenol–water dimer described in the main text (both the GIAO-B3LYP and GIAO-MP2 calculations were performed with the 6-311++G(2d,2p) basis set for the MP2/aug-cc-pVTZ geometry).

distance between the oxygens (in picometers)	angle (in radians) between $\overrightarrow{\xi_3}$ and the line connecting the oxygens		σ^{iso} (in ppm)	
	GIAO-B3LYP	GIAO-MP2	GIAO-B3LYP	GIAO-MP2
261.0	0.1407	0.1392	21.7608	21.7245
265.5	0.1429	0.1412	22.2620	22.2250
270.0	0.1452	0.1432	22.7143	22.6754
274.5	0.1474	0.1451	23.1222	23.0803
279.0	0.1496	0.1470	23.4890	23.4434
283.5	0.1518	0.1488	23.8209	23.7711
288.0	0.1539	0.1505	24.1205	24.0661
292.5	0.1560	0.1523	24.3910	24.3317
297.0	0.1580	0.1539	24.6355	24.5709
301.5	0.1600	0.1555	24.8573	24.7873
306.0	0.1619	0.1570	25.0581	24.9828
310.5	0.1638	0.1584	25.2391	25.1590
315.0	0.1656	0.1598	25.4039	25.3190
319.5	0.1674	0.1612	25.5538	25.4639
324.0	0.1691	0.1625	25.6908	25.5951
328.5	0.1707	0.1637	25.8160	25.7139
333.0	0.1723	0.1649	25.9313	25.8220
337.5	0.1738	0.1660	26.0367	25.9203
342.0	0.1753	0.1671	26.1327	26.0098
346.5	0.1767	0.1681	26.2201	26.0912
351.0	0.1781	0.1691	26.3002	26.1659

For the calculated ^{1}H CST of the H1 site of malonic acid, with the eigenvalues σ_{11}, σ_{22}, σ_{33} $(\sigma_{11} \leq \sigma_{22} \leq \sigma_{33})$ and their associated eigenvectors $\overrightarrow{\chi_1}, \overrightarrow{\chi_2}, \overrightarrow{\chi_3}$, it is convenient to employ the reference frame given by the vectors $\vec{p}, \vec{q}, \vec{r}$ that are defined as follows: $\vec{p}$ is a normal vector to the O1–C2–O2 plane; $\vec{q}$ is a vector which is perpendicular to the O1–H1 bond vector and which lies in the O1–C2–O2 plane; $\vec{r}$ is a vector parallel to the O1–H1 bond vector. The angles between the respective vectors from $\{\overrightarrow{\chi_1}, \overrightarrow{\chi_2}, \overrightarrow{\chi_3}\}$ and $\{\vec{p}, \vec{q}, \vec{r}\}$ sets are given by $A = \cos^{-1} B$; using the shorthand notation (a, b) for the absolute value of the scalar product of the vectors $\vec{a}$ and $\vec{b}$: $|\vec{a} \cdot \vec{b}|$, the matrix B is

$$B = \begin{bmatrix} (\chi_1, p) & (\chi_2, p) & (\chi_3, p) \\ (\chi_1, q) & (\chi_2, q) & (\chi_3, q) \\ (\chi_1, r) & (\chi_2, r) & (\chi_3, r) \end{bmatrix}$$

For the PW-PBE optimized structure with $\{\vec{p}, \vec{q}, \vec{r}\}$:

0.9975	0.0560	0.0425
-0.0203	0.8079	-0.5889
-0.1482	0.5801	0.8009

and with the GIPAW-PBE $\{\overrightarrow{\chi_1}, \overrightarrow{\chi_2}, \overrightarrow{\chi_3}\}$:

0.9830	0.0948	0.1570
-0.0162	0.8975	-0.4406
-0.1827	0.4306	0.8838

the elements of the matrix A are (in degrees):

7	89	83
88	10	80
88	80	10

This means $\overrightarrow{\chi_1}$ is approximately perpendicular to the O1–C2–O2 plane, $\overrightarrow{\chi_2}$ is approximately perpendicular to the O1–H1 bond, and $\overrightarrow{\chi_3}$ is almost collinear with the O1–H1 bond.

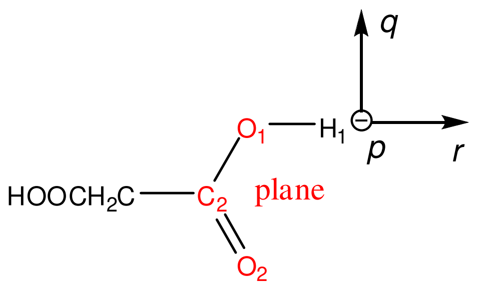

For the calculated ^{1}H CST of the H2 site of malonic acid, with the eigenvalues $\sigma_{11}, \sigma_{22}, \sigma_{33}$ $(\sigma_{11} \leq \sigma_{22} \leq \sigma_{33})$ and their associated eigenvectors $\overrightarrow{\chi_1}, \overrightarrow{\chi_2}, \overrightarrow{\chi_3}$, it is convenient to employ the reference frame given by the vectors $\vec{p}, \vec{q}, \vec{r}$ that are defined as follows: $\vec{p}$ is a normal vector to the O3–C3–O4 plane; $\vec{q}$ is a vector which is perpendicular to the O4–H2 bond vector and which lies in the O3–C3–O4 plane; $\vec{r}$ is a vector parallel to the O4–H2 bond vector. The angles between the respective vectors from $\{\overrightarrow{\chi_1}, \overrightarrow{\chi_2}, \overrightarrow{\chi_3}\}$ and $\{\vec{p}, \vec{q}, \vec{r}\}$ sets are given by $A = \cos^{-1} B$; using the shorthand notation (a, b) for the absolute value of the scalar product of the vectors $\vec{a}$ and $\vec{b}$: $|\vec{a} \cdot \vec{b}|$, the matrix B is

$$B = \begin{bmatrix} (\chi_1, p) & (\chi_2, p) & (\chi_3, p) \\ (\chi_1, q) & (\chi_2, q) & (\chi_3, q) \\ (\chi_1, r) & (\chi_2, r) & (\chi_3, r) \end{bmatrix}$$

For the PW-PBE optimized structure with $\{\vec{p}, \vec{q}, \vec{r}\}$:

-0.1558	0.8232	0.5460
0.9870	0.1516	0.0530
-0.0476	0.5914	-0.8050

and with the GIPAW-PBE $\{\overrightarrow{\chi_1}, \overrightarrow{\chi_2}, \overrightarrow{\chi_3}\}$:

-0.1709	0.7812	0.6005
0.9613	0.2658	-0.0723
0.2161	-0.5649	0.7964

the elements of the matrix A are (in degrees):

4	88	86
89	10	80
89	80	10

This means $\overrightarrow{\chi_1}$ is approximately perpendicular to the O3–C3–O4 plane, $\overrightarrow{\chi_2}$ is approximately perpendicular to the O4–H2 bond, and $\overrightarrow{\chi_3}$ is almost collinear with the O4–H2 bond.

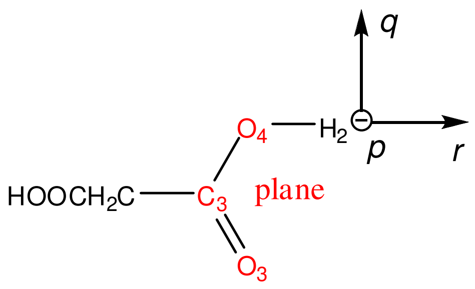

For the calculated ^{1}H CST of the H3 site of malonic acid, with the eigenvalues σ_{11}, σ_{22}, σ_{33} $(\sigma_{11} \leq \sigma_{22} \leq \sigma_{33})$ and their associated eigenvectors $\overrightarrow{\chi_1}, \overrightarrow{\chi_2}, \overrightarrow{\chi_3}$, it is convenient to employ the reference frame given by the vectors $\vec{p}, \vec{q}, \vec{r}$ that are defined as follows: $\vec{p}$ is a normal vector to the H3–C1–C3 plane; $\vec{q}$ is a vector which is perpendicular to the C1–H3 bond vector and which lies in the H3–C1–C3 plane; $\vec{r}$ is a vector parallel to the C1–H3 bond vector. The angles between the respective vectors from $\{\overrightarrow{\chi_1}, \overrightarrow{\chi_2}, \overrightarrow{\chi_3}\}$ and $\{\vec{p}, \vec{q}, \vec{r}\}$ sets are given by $A = \cos^{-1} B$; using the shorthand notation (a, b) for the absolute value of the scalar product of the vectors $\vec{a}$ and $\vec{b}$: $|\vec{a} \cdot \vec{b}|$, the matrix B is

$$B = \begin{bmatrix} (\chi_1, p) & (\chi_2, p) & (\chi_3, p) \\ (\chi_1, q) & (\chi_2, q) & (\chi_3, q) \\ (\chi_1, r) & (\chi_2, r) & (\chi_3, r) \end{bmatrix}$$

For the PW-PBE optimized structure with $\{\vec{p}, \vec{q}, \vec{r}\}$:

-0.3769	-0.8065	-0.4555
0.2058	0.4066	-0.8901
0.9031	-0.4293	0.0127

and with the GIPAW-PBE $\{\overrightarrow{\chi_1}, \overrightarrow{\chi_2}, \overrightarrow{\chi_3}\}$:

0.4276	0.6609	0.6168
0.4498	0.4363	-0.7793
-0.7841	0.6106	-0.1107

the elements of the matrix A are (in degrees):

13	80	82
79	15	79
84	78	14

This means $\overrightarrow{\chi_1}$ is approximately perpendicular to the H3–C1–C3 plane, $\overrightarrow{\chi_2}$ is approximately perpendicular to the C1–H3 bond, and $\overrightarrow{\chi_3}$ is almost collinear with the C1–H3 bond.

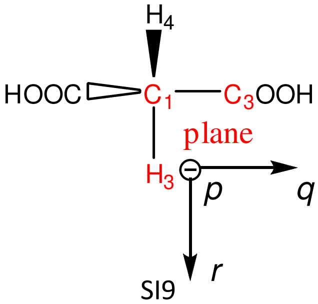

For the calculated ^{1}H CST of the H4 site of malonic acid, with the eigenvalues σ_{11}, σ_{22}, σ_{33} $(\sigma_{11} \leq \sigma_{22} \leq \sigma_{33})$ and their associated eigenvectors $\overrightarrow{\chi_1}, \overrightarrow{\chi_2}, \overrightarrow{\chi_3}$, it is convenient to employ the reference frame given by the vectors $\vec{p}, \vec{q}, \vec{r}$ that are defined as follows: $\vec{p}$ is a normal vector to the H4–C1–C3 plane; $\vec{q}$ is a vector which is perpendicular to the C1–H4 bond vector and which lies in the H4–C1–C3 plane; $\vec{r}$ is a vector parallel to the C1–H4 bond vector. The angles between the respective vectors from $\{\overrightarrow{\chi_1}, \overrightarrow{\chi_2}, \overrightarrow{\chi_3}\}$ and $\{\vec{p}, \vec{q}, \vec{r}\}$ sets are given by $A = \cos^{-1} B$; using the shorthand notation (a, b) for the absolute value of the scalar product of the vectors $\vec{a}$ and $\vec{b}$: $|\vec{a} \cdot \vec{b}|$, the matrix B is

$$B = \begin{bmatrix} (\chi_1, p) & (\chi_2, p) & (\chi_3, p) \\ (\chi_1, q) & (\chi_2, q) & (\chi_3, q) \\ (\chi_1, r) & (\chi_2, r) & (\chi_3, r) \end{bmatrix}$$

For the PW-PBE optimized structure with $\{\vec{p}, \vec{q}, \vec{r}\}$:

-0.6028	0.6365	0.4811
-0.3966	0.2842	-0.8729
-0.6924	-0.7170	0.0811

and with the GIPAW-PBE $\{\overrightarrow{\chi_1}, \overrightarrow{\chi_2}, \overrightarrow{\chi_3}\}$:

0.7851	-0.5673	-0.2485
0.3229	0.0326	0.9459
-0.5285	-0.8229	0.2088

the elements of the matrix A are (in degrees):

17	74	84
75	19	78
81	80	13

This means $\overrightarrow{\chi_1}$ is approximately perpendicular to the H4–C1–C3 plane, $\overrightarrow{\chi_2}$ is approximately perpendicular to the C1–H4 bond, and $\overrightarrow{\chi_3}$ is almost collinear with the C1–H4 bond.

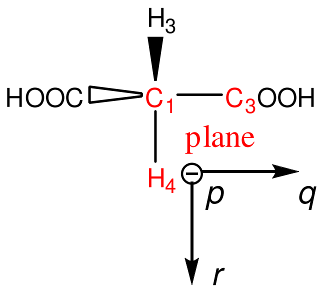

Supplement: Supplementary file 1 [file molecules-24-01731-s001.pdf]
